# Left Atrial Mural Thrombosis and Hemopericardium in a Dog with Myxomatous Mitral Valve Disease

**DOI:** 10.3390/vetsci8060112

**Published:** 2021-06-17

**Authors:** Domenico Caivano, Maria Chiara Marchesi, Francesco Birettoni, Elvio Lepri, Francesco Porciello

**Affiliations:** Department of Veterinary Medicine, University of Perugia, Via San Costanzo 4, 06126 Perugia, Italy; maria.marchesi@unipg.it (M.C.M.); elvio.lepri@unipg.it (E.L.); francesco.porciello@unipg.it (F.P.)

**Keywords:** echocardiography, left atrial rupture, mitral valve disease, mural thrombosis, pericardial effusion

## Abstract

A 14-year-old mixed-breed dog with a 2-year history of myxomatous mitral valve disease was examined for collapse and lethargy. At the presentation, pale oral mucous membranes, rapid and weak femoral pulses, and muffled heart sounds with a moderate left apical systolic murmur were revealed. Echocardiographic examination showed pericardial effusion with organized echogenic material originating from the left atrial wall. Tamponade of the right atrium and severe left atrial enlargement were also observed. Multiple views of the left atrium and left auricle allowed to visualize a hyperechoic mass adherent to the endocardium of the left atrial wall. Contrast-enhanced ultrasonography study allowed to rule out active intrapericardial hemorrhages, and echo-guided pericardiocentesis was performed. No recurrence of pericardial effusion was observed, but the dog suddenly died after 10 days. The postmortem examination confirmed multifocal left atrial thrombosis attached to the endomyocardial tears.

## 1. Introduction

Left atrial rupture has been previously reported as an uncommon complication of severe myxomatous mitral valve disease (MMVD) in dogs [1,2,3,4,5,6,7]. The etiology of left atrial rupture in dogs affected by MMVD can be multifactorial, however, endocardial splitting represents a predisposing factor in the development of this clinical condition [1,5,6,7]. Endocardial splitting can variably extend in depth into the myocardium with spontaneous tear of the endomyocardium [1,5,6,7]. Therefore, dogs with endocardial splitting can develop complete endomyocardial rupture with hemopericardium [1,2,3,4,5,6,7]. Conversely, an incomplete endocardial tear can result in intracardiac bleeding and thrombus formation, as previously reported in a dog [8].

Rupture of the left atrial wall results in a rapid accumulation of blood within the pericardium with clinical signs of cardiac tamponade and cardiogenic shock [1]. Pericardiocentesis may be performed to resolve cardiac tamponade, but in the case of ongoing hemorrhage, this can worsen the bleeding as a consequence of relieving the pericardial pressure during the fluid removal. Contrast-enhanced ultrasonography (CEUS) has been used for improving the detection of active pericardial hemorrhages during cardiac rupture in humans [9,10].

The present report describes a rare case of atrial mural thrombosis and hemopericardium in a dog affected by MMVD. Additionally, we describe the use of CEUS to rule out active intrapericardial hemorrhages from left atrial tears.

## 2. Case Presentation

**Figure 1:**
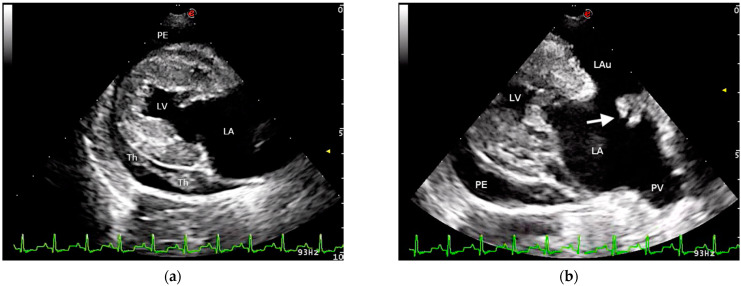
Conventional echocardiography at the presentation: (**a**) Right parasternal long-axis view showing pericardial effusion (PE) with hyperechoic density suggestive of a thrombus (Th). (**b**) Modified left parasternal long-axis view optimized to visualize the left atrium (LA) and auricle (LAu). A hyperechoic structure (arrow), adherent to the left atrial wall and consistent with a thrombus, is evident. LV, left ventricle; PV, pulmonary vein.

A 14-year-old, 5.5 kg, mixed-breed dog with a 2-year history of MMVD was presented to our hospital for collapse and lethargy. Conventional heart failure therapy (furosemide 2 mg/kg twice a day, orally; benazepril 0.5 mg/kg once a day, orally; pimobendan 0.25 mg/kg twice a day orally) had been initiated 10 months prior. Physical examination revealed pale oral mucous membranes, rapid and weak femoral pulses, and muffled heart sounds with a moderate left apical systolic murmur. Mild regenerative anemia (Hct 35%, reference limits 37–55%) with thrombocytopenia (PLT 180 × 10^3^/µL, reference intervals 200–500 × 10^3^/µL) were detected on CBC. Serum biochemical analysis revealed high BUN (90 mg/dL, reference limits 20–40 mg/dL) and creatinine (2.2 mg/dL, reference limits 1–2 mg/dL) concentrations. Electrocardiographic examination showed sinus tachycardia (170 bpm) with sporadic atrial premature beats. Two-dimensional echocardiography identified severe left atrial enlargement (left-atrial-to-aortic ratios = 3), left ventricular dilation (diastolic and systolic left ventricular internal diameter were 3.82 cm and 1.61 cm, respectively), marked thickening, and prolapse of the leaflets of both atrioventricular valves. Moderate pericardial effusion with right atrium collapse was also observed. An organized echogenic material originating from the wall of the left atrium was evident within the pericardial space (Figure 1). Multiple views of the left atrium and left auricle revealed a hyperechoic mass adherent to the atrial wall (Figure 1). Severe mitral and mild tricuspid valve regurgitation was demonstrated by color Doppler echocardiography. Velocity of tricuspid valve regurgitation was 2.76 m/sec (pressure gradient = 30.5 mmHg). Mild abdominal effusion with caudal vena cava distention and hepatic venous congestion were also identified on ultrasonography.

**Figure 2:**
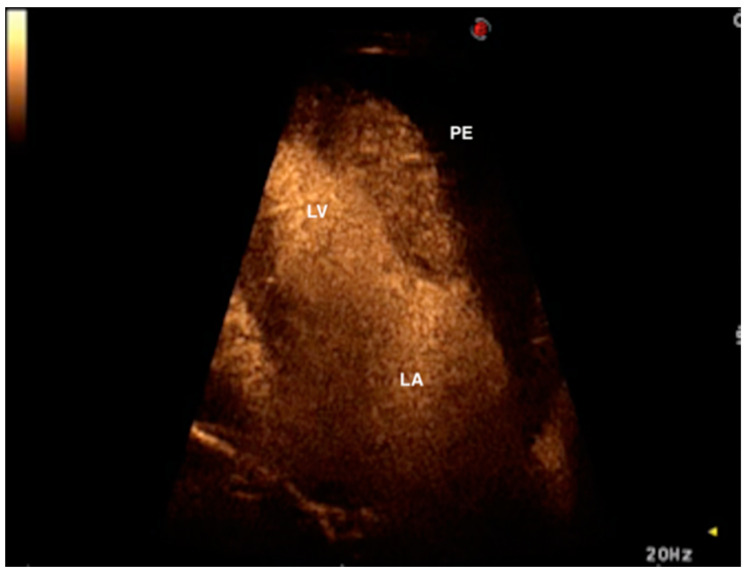
Contrast-enhanced echocardiographic image (left apical 4-chamber view) showing no evidence of microbubbles in the pericardial space after the complete opacification of the cardiac chambers. PE, pericardial effusion; LV, left ventricle; LA, left atrium.

A diagnosis of severe MMVD, hemopericardium with cardiac tamponade secondary to left atrial rupture, and left atrial thrombus was made. To enhance the visualization of active intrapericardial bleeding, CEUS was performed using an ultrasound machine (MyLab Class C, Esaote, Florence, Italy) equipped with a contrast-tuned imaging technology module (CnTI^TM^, Esaote, Florence, Italy). A bolus (0.05 mL/kg) of sulfur hexafluoride microbubbles stabilized by a phospholipid shell (SonoVue^®^, Bracco, Milano, Italy) was intravenously infused by hand as previously described [11]. The CEUS study demonstrated no evidence of microbubbles in the pericardial space after the complete opacification of the left atrium (Figure 2). This finding was suggestive of the absence of active intrapericardial hemorrhages, thus echo-guided pericardiocentesis was performed, yielding 35 mL of hemorrhagic fluid. No recurrence of hemopericardium was noted over the hospitalization period (3 days) and the dog was discharged with furosemide (2 mg/kg twice a day, orally), benazepril (0.5 mg/kg once a day, orally), pimobendan (0.25 mg/kg twice a day, orally), and clavulanate-amoxycillin (12.5 mg/kg twice a day, orally). In addition, clopidogrel (2 mg/kg once a day, orally) was prescribed to further inhibit the enhancement of the left atrial thrombus.

**Figure 3:**
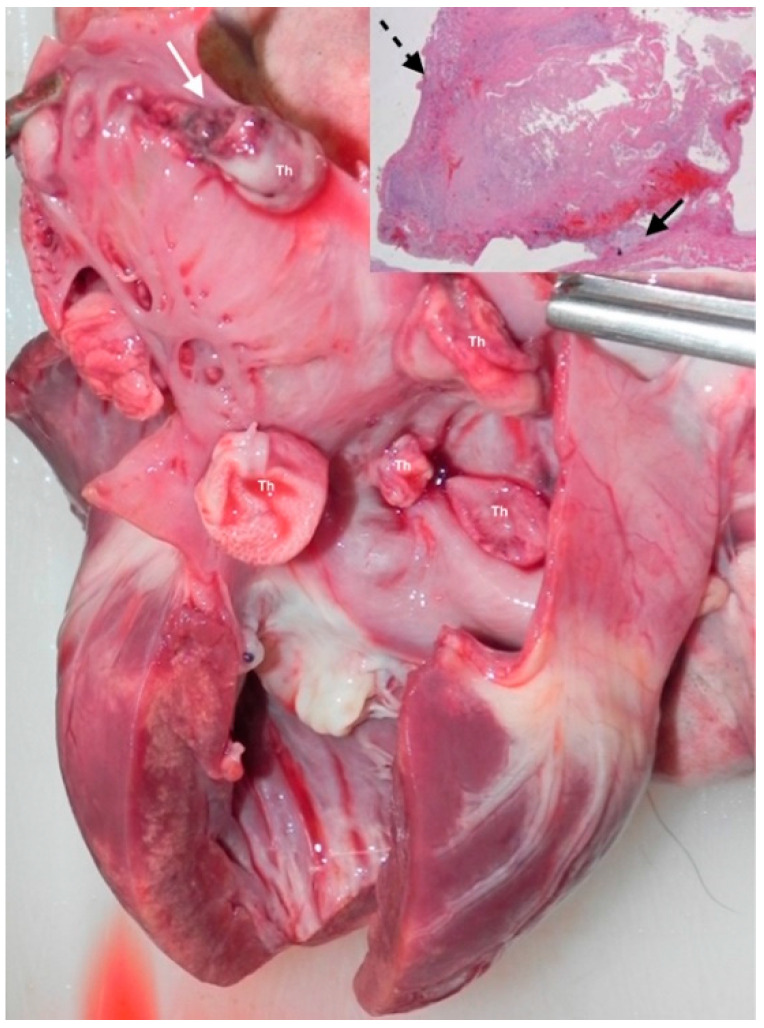
Necroscopy imaging. Several, up to 1 cm, pink, pedunculated masses (thrombi) adherent to the mural endocardium of the interatrial septum and left auricle. Note the hemorrhages around the base in the upper thrombus (white arrow). Mitral septal (anterior) leaflet is thickened and distorted, and in the myocardium multifocal to coalescing whitish areas (necrosis with calcifications). Insert: pedunculated thrombus (dashed arrow) attached to the endocardial surface of the auricle (black arrow); Hematoxylin and eosin, low magnification. Th, thrombus.

Seven days after the discharge, the dog suddenly died, and a complete necroscopy was performed. At the opening of the thoracic cavity, the pericardial sac was thickened and edematous; the pericardial cavity contained only a small amount of serosanguineous fluid; in the left atrial lumen, multiple pedunculated 1–2 cm, red-pink, smooth, soft masses were present, adherent to interatrial septum, atrial, and auricular wall; at the base of some masses a red discoloration was present (Figure 3). In the myocardium, multifocal poorly defined whitish areas were present. Mitral and, to a lesser extent, tricuspidal leaflets were thickened and distorted. All the other examined tissues had no gross lesions, including brain. Histologically the main findings were hepatic diffuse centrolobular hemorrhagic necrosis, multifocal tubular renal necrosis. In the heart, apart from severe myxoid degeneration of atrioventricular valves, multifocal to coalescing severe acute myocardial necrosis with calcifications was present, involving about 30–40% of myocardium, both in the left and right ventricular free wall and interventricular septum; the left atrium contained multifocal mural thrombosis (Figure 3).

## 3. Discussion

The case described in this report represents the first description of antemortem diagnosis of atrial mural thrombosis associated with hemopericardium in a dog affected by MMVD. Mural thrombosis and hemopericardium due to left atrial rupture can occur in dogs with severe MMVD, but these have not been previously reported as concomitant complications. Thrombus formation is an uncommon complication of cardiac diseases in dogs. Intracardiac thrombus in the left atrium has been reported in dogs with atrial fibrillation [12], secondary to blunt cardiac injury [13], or as a consequence of myocarditis [14]. Recently, thrombi attached to partial left atrial endocardial tears have been described in a dog with MMVD and myocardial infarct [8]. Pericardial effusion is usually associated with tumors or idiopathic pericardial effusion in dogs; less commonly, it is secondary to coagulopathy, local infections, congestive heart failure, or left atrial rupture [1,2,3,4,5,6,7,15,16,17,18,19,20]. Left atrial tears can occur in the lateral wall of the left atrium secondary to MMVD and cause pericardial effusion, cardiac tamponade, and sudden death in dogs [1,2,3,5]. Buchanan and Kelly described left atrial tears in a first report of 22 dogs and 7 of these showed pericardial effusion [1]. Rarely, acquired atrial septal defects secondary to MMVD have been reported in dogs: fossa ovalis can be a site of tear as a consequence of mechanical trauma or increased atrial pressure [21,22]. The etiology of the left atrial tears in dogs affected by MMVD is unclear: Increased left atrial volume/pressure and mechanical trauma from high velocity mitral regurgitation jet represent predisposing factors for spontaneous tears [1,5,6,7]. The dog described in this report presented atrial thrombosis and hemopericardium secondary to left atrial rupture: Multiple left atrial tears with variable depths were able to cause both intracardiac and intrapericardial hemorrhages. No acquired atrial defect was visualized in the interatrial septum on echocardiography and necroscopy. In the present case, the death of the dog was attributed to severe myocardial necrosis, inducing severe hypotension and tissue hypoxia that led to centrilobular hepatic necrosis and renal tubular necrosis, both presumed to be hypoxic. Causes of severe myocardial necrosis in dogs include thromboembolic events, which is unlikely to cause extensive multifocal necrosis, as well as toxic or nutritional. None of these causes were identified in this case, and the exact cause of myocardial necrosis remains unexplained.

Transthoracic echocardiography is a noninvasive, reliable, and fast-imaging technique that can be useful for diagnosis of pericardial diseases, cardiac masses, myocardial and valvular diseases. In our report, transthoracic echocardiography allowed to demonstrate pericardial effusion and cardiac tamponade secondary to left atrial rupture. Moreover, echocardiographic images of the pericardial effusion were characterized by an organized echogenic material, originating from the wall of the left atrium, suggestive of a thrombus. This echocardiographic feature associated to left ventricular and atrial enlargement, and mitral valve thickening and regurgitation, have been reported as specific echocardiographic criteria for antemortem diagnosis of left atrial rupture secondary to MMVD [4,5,7]. In the dog of this report, transthoracic echocardiography also allowed to visualize a hyperechoic mass adherent to the left atrial wall. Differential diagnosis of the left atrial masses included neoplasia and thrombus. Cardiac tumors are extremely rare (0.19%) in dogs, and sporadically, tumors involve the left-sided heart chambers [23]. Based on the echocardiographic appearance of the intracardiac mass in the reported dog, a presumptive diagnosis of left atrial thrombus was made. Postmortem examination confirmed multifocal left atrial thrombosis attached to the endomyocardial tears. Our findings are similar to the previous case report of Sleeper et al. [8]. Indeed, these authors reported an antemortem diagnosis of myocardial infarct presumed secondary to left atrial thrombi, which were confirmed with necropsy [8]. Diagnosis of presumptive left atrial thrombosis was made on the ultrasonographic appearance and anatomic position of the intracardiac masses [8]. Moreover, these masses appeared to adhere to the LA wall on echocardiography [8], as observed on echocardiography and necroscopy in our dog.

Left atrial rupture can lead to cardiovascular instability involving pericardial effusion, cardiac tamponade, reduced preload, and low cardiac output. If cardiac tamponade and clinical signs of hemodynamic compromise are present, the drainage of the effusion from the pericardial space is necessary, but pericardial effusion can recur because of relieving the pericardial pressure during the fluid removal. In humans, CEUS have been used for improving the detection of active intrapericardial hemorrhages due to cardiac rupture [9,10]. Evidence of microbubbles in the pericardial space was considered suggestive of a myocardium tear in patients with pericardial effusion [9,10]. Contrast-enhanced ultrasonography is an imaging modality that improves the diagnostic accuracy of conventional ultrasonography by increasing the intensity of blood-pool echo signals in vessels and heart through intravenous injection of stabilized gas-filled microbubbles as a contrast agent [11,24]. In our case, CEUS study demonstrated no evidence of microbubbles in the pericardial space after the complete opacification of the left atrium. This feature was suggestive of no active intrapericardial hemorrhages and pericardiocentesis was performed without recurrence of hemopericardium. However, CEUS can be useful for improving the detection of active intrapericardial hemorrhages, but cannot exclude a recurrence of hemopericardium because of relieving the pericardial pressure during the fluid removal. Further studies are needed to discern the value of the CEUS in dogs with left atrial rupture. Additionally, the use of CEUS could open up new perspectives for assessing other cardiac diseases characterized by bleeding (e.g., tumors or pericarditis), using this advanced ultrasonographic technique.

A recent consensus statement regarding rational use of antithrombotic drugs in dogs and cats recommended the administration of anticoagulants in venous thrombosis and antiplatelet drugs in arterial thrombosis, although no recommendation for using these drugs in combination was stated because of low clinical evidence [25]. A previous study reported no anticoagulant treatment in dogs with left atrial rupture secondary to MMVD because no clinical suspicion of thromboembolic disease could be advanced in these dogs [7]. In the dog described in this report, left atrial thrombosis was suspected and an antiplatelet drug (clopidogrel) was used to limit further enhancement of the left atrial thrombus. Although no recurrence of hemopericardium was observed in our dog, antiplatelet therapy could increase the bleeding risk in dogs with left atrial rupture. Therefore, clinicians should carefully consider the use of antithrombotic drugs in dogs with left atrial rupture and thrombosis, assessing the risk of bleeding and possible recurrences of the hemopericardium.

## 4. Conclusions

Left atrial tears can occur in dogs with severe MMVD, and clinical presentation can be characterized by hemopericardium, intracardiac thrombosis, or both, as described in the present case report. Moreover, CEUS could be a useful diagnostic tool in dogs with left atrial rupture to rule out active intrapericardial hemorrhages.
